# Improving coastal water level estimation by merging nadir-only satellite altimetry data into a hydrodynamic model

**DOI:** 10.1007/s10661-026-15166-8

**Published:** 2026-03-14

**Authors:** Soelem Aafnan Bhuiyan, Andre De Souza De Lima, Tyler Miesse, Martin Henke, Celso Ferreira, Viviana Maggioni

**Affiliations:** 1 Department of Civil, Environmental and Infrastructure Engineering, George Mason University, Fairfax, VA USA; 2 Pacific Northwest National Laboratory, Richland, WA USA

**Keywords:** ADCIRC, SWOT, Storm surge, Data assimilation, Altimetry

## Abstract

**Abstract:**

Providing robust real-time flood warnings is of paramount importance to coastal communities. Although state-of-the-art hydrodynamic models are capable of robustly predicting coastal water levels (CWL), unresolved drivers affecting water level fluctuations are often not represented by the model governing equations. This work evaluates a novel method to improve the performance of the ADvanced CIRCulation (ADCIRC) hydrodynamic model by assimilating observations from four nadir-only satellite altimetry missions with a set of National Oceanic and Atmospheric Administration (NOAA) gauge stations located across the entire U.S. East Coast. Two different types of simulations were performed - open loop (OL) and data assimilation (DA). Five different simulations were performed in which four different satellite altimetry observations were assimilated individually and under two different scenarios - with and without considering the data quality flags. Results indicate that, despite their limited spatial coverage, merging nadir-only observations into ADCIRC from thenadir altimeter of the Surface Water and Ocean Topography (SWOT) can improve the model performance at 76% of the gauge locations, whereas Sentinel-6 improves it at 73% of the total locations, Jason-3 at 74%, and SARAL at 21%. Furthermore, combining observations from SWOT-nadir, Jason-3, and Sentinel-6 can improve the ADCIRC performance at more than 80% of the gauge locations for a 107-day simulation. Nadir-only satellite altimetry observations can be useful for improving the model performance even if flagged as “poor quality” near the coast. When the flagged data are disregarded, SWOT can improve ADCIRC at 78% of the gauge locations, Sentinel-6 at 73%, Jason-3 at 53%, and SARAL at 21%. The ability to improve the model simulations largely depends on the availability of a nearby satellite overpass. Therefore, model performance can be further enhanced if satellite observations are available during a storm surge event, stressing the importance of frequent satellite overpasses.

**Research highlights:**

- Nadir-only satellite altimetry improves storm surge model performance.
- Model skill increases when overpasses capture surge events.
- Multi-mission altimetry assimilation yields the highest overall accuracy.

**Supplementary Information:**

The online version contains supplementary material available at 10.1007/s10661-026-15166-8.

## Introduction

Due to intensified sea-level rise (SLR) and recurrent extreme weather events, coastal communities are becoming more vulnerable to coastal flooding (Wahl & Chambers, 2015). Projections indicate a potential global mean SLR exceeding 1 m within this century, exacerbating flood risk due to reduced freeboard between high tides and flood stages (Arns et al., 2020; Boumis et al., 2023; Buchanan et al., 2017; Dangendorf et al., 2019; Hunter, 2012; Intergovernmental Panel on Climate Change (IPCC), 2023; Nicholls et al., 2021; Palmer et al., 2020). The combination of more severe storms and rising sea levels is expected to amplify storm surge risks in coastal regions, increasing the hazard potential in U.S. coastal areas (Dinan, 2017; Lin et al., 2012). In recent years, both coastal and estuarine areas have been experiencing record-breaking flooding from hurricanes such as Katrina, Irene, Ida, and Sandy. Current estimates suggest that without intervention, 33 U.S. cities will experience unprecedented coastal flooding by 2050, and this number would increase to 90 in 2100 (S. A. Kulp & Strauss, 2019; S. Kulp & Strauss, 2017). Monitoring coastal water level (CWL) is essential not only for navigational safety but also to assess and forecast sea-level changes and their impact on coastal zones, thereby informing mitigation efforts and adaptation strategies (Moftakhari et al., 2024; Palanisamy et al., 2014; Tebaldi et al., 2021). Accurate estimation of CWL thus remains of utmost importance.

CWL is affected by various factors, e.g., tides, wind, storm surges, nearshore elevation, and rainfall. Extreme events such as hurricanes can cause significant atmospheric fluctuations, and seasonal anomalies can contribute to those fluctuations. Additionally, changes in tidal amplitudes can also play a role in CWL variations (Cheng et al., 2017). Other drivers, e.g., Atlantic Meridional Overturning Circulation (AMOC), have been observed to induce fluctuations in coastal sea levels. For instance, higher sea levels on the U.S. east coast have been correlated with AMOC (Ezer, 2015; Robson et al., 2014; Smeed et al., 2014). Ezer and Updyke (2024) argued that SLR in Chesapeake Bay is linked to AMOC weakening and increased river discharge. Gulf Stream changes can cause fluctuations of several tens of centimeters in sea level, as seen during Hurricane Matthew, where a 30–40 cm rise was associated with a 30% reduction in Gulf Stream transport (Andres et al., 2013; Calafat et al., 2018; Hong et al., 2000). Failing to accommodate these fluctuations in a coastal hydrodynamic model affects model performance.

There are a number of ways to monitor and estimate CWL. Tide gauges have been considered a reliable source of information along the coastlines for more than a century. A well-known example is the Pier & Tide Gauge House in the San Francisco Bay. However, many limitations exist in observing water level using tide gauges (Lobeto et al., 2018). First off, they have limited spatial coverage; second, they are heavily influenced by local geography; and third, they are often affected by vertical land movement (Peltier, 2001; Wöppelmann & Marcos, 2016). Another source of CWL data is satellite altimetry. Satellites have recorded sea surface height (SSH) for nearly three decades. In the open ocean, where the ubiquitous measurement of sea level with buoys is a logistically challenging task, satellite altimetry has been proven to be advantageous. Satellites have been a consistent and trustworthy source of mean sea level data and sea level rise trends (Church et al., 2013). Although satellite altimetry missions have been proven to be useful in the coastal zones by robustly complementing the information provided by tide gauges (Vinogradov & Ponte, 2011), their quality is known to degrade near coastline (Birol et al., 2017; Idžanović et al., 2017).

Studies over the past two decades have provided important information on the rapid deterioration in data accuracy near the shorelines, especially within 30–50 km (Anzenhofer, 1999; Birol et al., 2017; Gommenginger et al., 2011). This phenomenon can generally be attributed to “land contamination” in the radar signal, where the interaction of the signal with the land surface makes its interpretation difficult (X. Deng & Featherstone, 2006; Y. Deng et al., 2010). Due to the inherent characteristics of microwave radar pulses, the SSH derived from satellite altimetry has to undergo a number of corrections (Andersen & Scharroo, 2011). Such corrections are both instrumental and geophysical (e.g., wet and dry tropospheric correction, ionospheric correction, and sea state bias) and are complicated by the vicinity of the coast, where the water is shallow (Andersen & Scharroo, 2011). Thus, along-track nadir altimetry data are often flagged as “bad” near coastlines. In coastal and nearshore regions, altimetry waveforms often do not conform to the Brown model assumptions, as waveforms can be corrupted by land returns, shallow-water effects, and rapidly changing wave conditions (Abessolo et al., 2023; Brown, 1977; Wang & Huang, 2021).

Retracking algorithms also play a key role in the accuracy of satellite altimetry in coastal areas (Mafi et al., 2024). Maximum likelihood estimator (MLE) algorithms such as MLE3 and MLE4 are commonly used to fit the model to the observed waveforms (Thibaut et al., 2010). These approaches differ in the number of model parameters: MLE3 estimates three parameters, while MLE4 estimates four. With a few exceptions, the majority of current altimetry missions use the MLE4 retracking algorithm by default, which is more suitable for coastal applications compared to MLE3. Sentinel-6 uses a numerical retracker (NR) on top of the default MLE4 retracker (Cadier et al., 2025). Standard MLE4 is also used by Jason-3 and SARAL (Tran et al., 2021). Additionally, the coarse orbital frequency of the satellites makes it difficult to observe rapidly varying processes such as storm surges. For example, Jason-3 and Sentinel-6 satellites have orbital repeat intervals of ~ 10 days, whereas SARAL has a repeat interval of 35 days. However, advancements in sophisticated radar technologies have significantly improved our ability to measure SSH and extended these improvements to coastal areas. These include advanced reprocessing techniques, better resolution in both the open ocean and coastal regions, and robust algorithms for data extraction from radar echoes (Stammer & Cazenave, 2017). These technologies have enabled more accurate characterization of ocean circulations, mesoscale and sub-mesoscale processes, and specific ocean features, such as eddies, baroclinic processes, major currents, and steric variations (Zawadzki & Ablain, 2016).

To address the limitations of tide gauge data and satellite altimetry data for estimating and predicting CWL, assimilating those observations into numerical models can be useful (Bhuiyan et al., 2024; Bij De Vaate et al., 2024). Numerical models are essential tools for estimating CWL, with significant accuracy improvements over the past decade (Fleming et al., 2008; Resio & Westerink, 2008). For instance, the ADvanced CIRCulation (ADCIRC) (Luettich & Westerink, n.d.) model provides reliable simulations of coastal dynamics with high spatial and temporal resolution by utilizing time-dependent, multidimensional free-surface circulation models (Blain et al., 1994; Deb & Ferreira, 2018; Viitak et al., 2021). Hydrodynamic models effectively integrate regularly gridded atmospheric forcing estimates, e.g., the European Center for Medium-Range Weather Forecasts (ECMWF) Reanalysis v5 (ERA5) (Hersbach et al., 2020), with coastal bathymetry and astronomical tides. Products such as the ECMWF atmospheric reanalysis dataset offer data-assimilated and validated atmospheric forcings, e.g., wind fields, for storm surge simulations (Westerink et al., 2008). Recent advancements in unstructured finite element mesh quality have further enhanced the accuracy of storm surge forecasts (Bilskie et al., 2020). The performance of these models, however, is contingent upon the quality of the unstructured mesh and meteorological forcings. Advancements in forcing data, specifically the wind data that act as the primary mechanism in generating storm surge, have improved hydrodynamic model performance in simulating extreme events (Cardone & Cox, 2009; Das et al., 2016). Thus, low-frequency unresolved drivers can now be primary contributors to model errors (Asher et al., 2019). These unresolved drivers include seasonal fluctuations in water level, large-scale oceanic circulations, rainfall runoff, etc.

This study merges satellite observations of coastal water height into ADCIRC with the objective of improving the estimation of CWL. We take advantage of the localized CWL observed by multiple satellite altimetry missions by directly assimilating them into ADCIRC. Data from four nadir-only altimetry missions are considered for locally correcting the ADCIRC simulations: Jason-3, Sentinel-6, SARAL, and the Surface Water and Ocean Topography (SWOT) mission. The performance of  the assimilated and non-assimilated simulations is compared against verified observations from the National Oceanic and Atmospheric Administration (NOAA) tide gauges along the U.S. East Coast. It is worth noting that during the 2023 Atlantic hurricane season, two notable instances of storm surge-induced coastal flooding occurred on the U.S. East Coast. One of them was caused by Tropical Storm Ophelia, which made landfall in North Carolina on September 23, 2023, and triggered flooding due to storm surge on the state’s eastern shore. Hurricane Idalia made landfall on the western coast of Florida on August 28, 2023, and caused up to 4 m of surge. Furthermore, we investigated the shift in model performance during these two surge events based on the availability of satellite overpasses.

## Methodology

### Model configuration

The study area consists of the eastern U.S. coast of the Atlantic Ocean and the Gulf of Mexico. ADCIRC (v55) is employed to simulate water levels in coastal and estuarine environments. Being a finite-element, shallow water model, ADCIRC simulates water levels at various scales. This model integrates astronomical tides and atmospheric wind and pressure fields to simulate water levels and velocities.

**Fig. 1:**
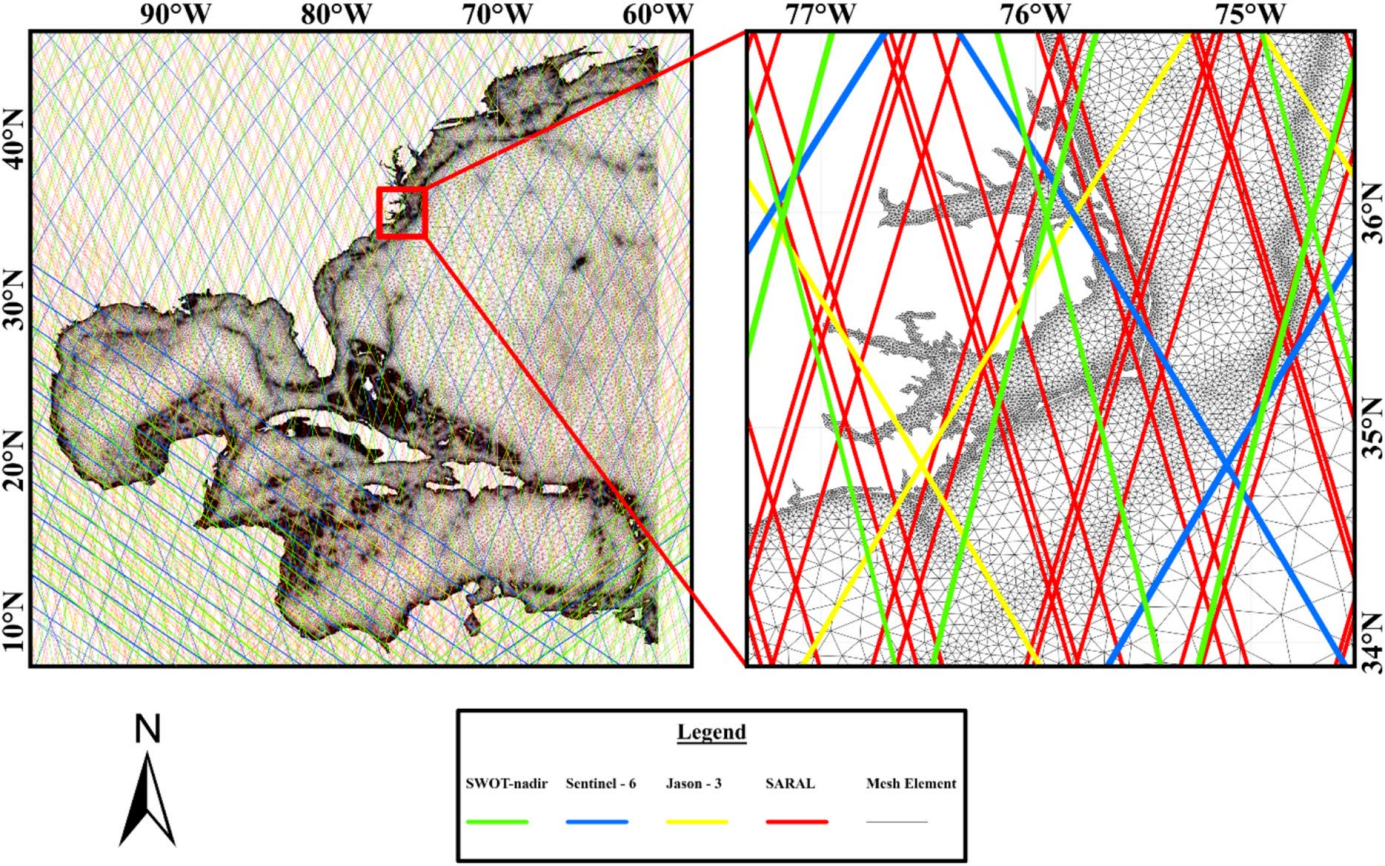
ADCIRC model mesh overlaid with the satellite tracks used in this study, with the red box highlighting the high resolution near the North Carolina coastline in the inset. The mesh has the highest resolution of 100 m near the U.S. coastline and the lowest resolution of 60 km

ADCIRC utilizes an unstructured grid to accurately represent topography, bathymetry, and both structural (e.g., weir) and non-structural features (e.g., friction) in the study region. The mesh for this study spans the entire U.S. East Coast, with a resolution as fine as 100 m near the coastline and a coarser resolution toward the ocean boundary, partially covering the western Atlantic Ocean (Fig. 1). Atmospheric forcing for ADCIRC was provided by the gridded ECMWF Re-Analysis 5 (ERA5) product (Hersbach et al., 2020). For simulating water level over a span of 107 days, two atmospheric forcing datasets were used: barometric pressure at mean sea level and wind velocity at 10 m above sea level. Hourly ERA5 atmospheric reanalysis data collected between July 16, 2023, and October 31, 2023, were used as forcings for ADCIRC simulations. Tidal forcings, derived from nine tidal constituents, including major semidiurnal and diurnal components, were based on the Oregon State University (OSU) TPXO9v2 global tidal database for the Chesapeake Bay and the U.S. Atlantic Ocean region (Egbert & Erofeeva, 2002). The model was cold-started on July 16, 2023, with tidal forcings and ERA5 atmospheric forcings. The first 16 days of the simulation, from July 16, 2023, to July 31, 2023, were used as a spin-up period to stabilize the simulation for high-frequency fluctuations such as astronomical tides. However, the SWOT regular sampling period did not start until July 10; thus, the earlier period was not included in the study. During this period, Hurricane Idalia made landfall on the Florida coast and tropical storms generated storm surge on the North Carolina coast.

### Satellite data

The four nadir-only satellite altimetry data used in this study are described next. SARAL is a radar altimeter operating in the Ka-band at 35.75 GHz, a higher frequency than traditional altimeters (Verron et al., 2015). The SARAL Ka-band pulse has a vertical resolution of 0.3 m (accuracy improves after applying retracking processes) and an 8 km-diameter footprint which is smaller than that of other similar missions (Verron et al., 2021). The satellite has a repeat cycle of 35 days and covers a global range between 81.5° N and 81.5° S. SARAL provides SSH products with both 1 Hz and 20 Hz frequencies, which enables precise monitoring of SSH, waves, and wind speeds. Jason-3 operates with a dual-frequency radar altimeter (POSEIDON-3B), working in the Ku-band (13.6 GHz) and C-band (5.3 GHz) (Desai, 2016). The altimeter’s primary mission is to monitor global mean sea level with a spatial resolution of about 2 km along the nadir track. The temporal resolution of Jason-3 is ~10 days. The satellite also includes an Advanced Microwave Radiometer (AMR) for atmospheric correction, a DORIS (Doppler Orbitography and Radiopositioning Integrated by Satellite) system for precise orbit determination, and a Laser Retroreflector Array (LRA) for satellite laser ranging. Sentinel-6 features the Poseidon-4 radar altimeter, operating at dual frequencies (Ku-band at 13.575 GHz and C-band at 5.3 GHz) to measure SSH with high precision of a few centimeters (SENTINEL-6, 2021). The altimeter footprint can range between 1and 10 km, depending on the wave height observed over the ocean. The satellite operates on a 10-day repeat cycle, covering the entire globe. Additionally, Sentinel-6 carries a Microwave Radiometer (AMR-C) for atmospheric water vapor correction, a GNSS Radio Occultation receiver for atmospheric profiling, a DORIS system, and a Laser Retroreflector for orbit determination and calibration.

The latest addition to the constellation, the SWOT mission, presents an opportunity to observe Earth’s surface water in wide swaths globally (Biancamaria et al., 2010; Morrow et al., 2019; Solander et al., 2016; SWOT, 2024). SWOT is equipped with a Ka-band interferometer (which operates at 35.75 GHz) with 60 km-wide swaths on two sides with ~ 10 km gap in between them for the nadir altimeter, a dual-frequency nadir altimeter, and a multi-frequency water vapor radiometer (Esteban-Fernandez et al., 2010; Fjortoft et al., 2014). This technology enables the measurement of water surface heights with a spatial resolution of 10–70 m for inland waters and up to 250 m for ocean waters. During its scientific observation phase, for the first 6 months after launch (December 15, 2022), SWOT had a revisit frequency of approximately 1 day. Afterward, during its regular sampling period, the revisit frequency varies from 3 to 21 days contingent upon latitude (Biancamaria et al., 2010), offering more frequent observations for particular regions compared to any existing altimetry missions. Moreover, SWOT provides an advantage with its non-sun-synchronous orbit, allowing for finer spatial resolution ocean topography measurements by the Ka-band interferometer than that of previous missions (Fu, 2009). Experiments with a constellation of two wide-swath altimeters similar to the dual-sided wide-swath altimetry of SWOT have demonstrated improvements in our understanding of ocean analysis and forecasting when compared to single wide-swath altimetry (Bonaduce et al., 2018; Turki et al., 2015). However, as this study focused only on nadir altimetry measurements, SWOT swath observations were not taken into account. The MLE4 retracker is available for the Level-2 Geophysical Data Record (GDR) for all four altimetry products used in this study. We have considered only 1-Hz data for assimilation and the default MLE4 retracker was used for all four products, i.e., no custom retracking algorithm was applied.

To retrieve instantaneous SSH comparable to the levels generated by ADCIRC and the gauge observations, satellite data must be corrected for a number of instrumental and geophysical errors. Satellites use microwave radar pulses to measure the height of water. As such pulses propagates through the atmosphere, interaction with water vapor slows down the speed of the electromagnetic waves. Thus, the range needs to be corrected for a number of geophysical errors. Furthermore, the satellite instrument altitude and the observed ocean surface height are measured above the reference ellipsoid. Thus, satellite products need to be converted with reference to the local geoid height to make them comparable to the gauge observations and ADCIRC simulations. The geoid used by the satellites is EGM-2008 (Pavlis et al., 2012). For this study, we have followed the Jason-3 product handbook for producing the Corrected Range of altimeter (*Jason-3 Products Handbook*, (Jason-3 n.d.), Chapter 4.2.1). Sea Level Anomaly (SLA) can be produced from Jason class altimetry products by applying standard geophysical corrections such as the Solid Earth Tides, Ocean Tides, Loading Tides, Pole Tides, and Dynamic Atmospheric Corrections (*Jason-3 Products Handbook*, n.d., Chapter 4.2.2). However, a few of these corrections (Ocean Tides, Loading Tides, Dynamic Atmospheric Corrections) are designed to remove high-frequency signals from the altimetry data, thereby producing long-term SLA data, which is more suitable for long-term averaged sea level variations. However, this study uses instantaneous sea level information recorded by satellite altimeters. ADCIRC simulates coastal water levels using astronomical tidal constituents, wind speed, and atmospheric pressure above sea level. The simulated water level generates water height with reference to a known datum, in this case, the Local Mean Sea Level (LMSL). Since we simulated water levels in reference to LMSL and did not produce long-term SLA, we have not considered the aforementioned corrections to retain the dynamic components of the altimetry products. The instantaneous SSH range (*R*_corr_) is therefore calculated as follows:

1 $${R}_{\mathrm{corr}}= {R}_{\mathrm{alt}}- {R}_{\mathrm{ocean}}-(SSB+ION+DTC+WTC+SET+PT)$$

where *R*_corr_ is the Corrected SSH Range; *R*_alt_ is the Satellite Altitude Above Reference Ellipsoid; *R*_ocean_ is the Ocean Surface Height Above Reference Ellipsoid; SSB is the Sea State Bias; ION is the Ionospheric Correction; DTC is the Dry Tropospheric Correction; WTC is the Wet Tropospheric Correction; SET is the Solid Earth Tide; and PT is Pole Tide.

For ADCIRC-simulated water level, mean sea level was used as the benchmark. The ADCIRC model used in this study is generated from the GEBCO-2024 Digital Elevation Model (DEM) (Weatherall et al., 2015) and NOAA NCEI Continuously Updated DEM (CUDEM), both of which use mean sea level and local mean sea level as vertical datums. Along with several other data, NOAA tide gauge observations are also retrievable from the mean sea level datum. As mean sea level can be considered roughly equal to the EGM-2008 geoid if not accounting for low-frequency sea level variations (Slobbe et al., 2012), we subtracted the geoid height from the satellite range, which otherwise is based on the reference ellipsoid height. When combined with the instrumental and geophysical corrections, the sea surface height (Eq. 2) translates to:

2 $$SSH=R_\mathrm{corr}-GEOID$$

where GEOID is the EGM-2008 Geoid Height Over Reference Ellipsoid. We have used NOAA tide gauges for testing the model simulation performance which are consistent with the water level generated by ADCIRC and observed by the altimeters as NOAA gauges are frequently corrected for vertical land motion and other localized vertical variations.

### The assimilation scheme

**Fig. 2:**
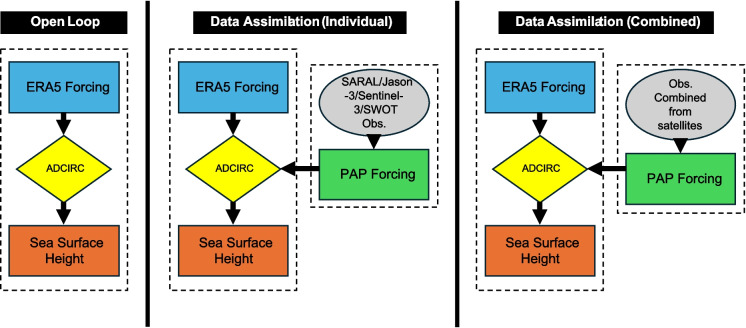
Workflow for ADCIRC simulations for the open loop (left) and the assimilation of satellite observations by PAP scheme (center and right)

The assimilation scheme applied in this study is Direct Insertion (DI) (Bhuiyan et al., 2024; Houser et al., 1998; Rahman et al., 2020). The DI scheme is the simplest available DA scheme that assumes no existing bias in the assimilated data and uses the direct application of observations into the model (Reichle, 2008). We generated and added localized corrections directly to the simulated water level based on observations from nadir-only satellite altimetry missions. The correction gradually varied in space and time and was applied to the water level field. Direct manipulation of the water level will not accomplish this by itself because a rapid barotropic adjustment would occur in the velocity field and the added water would simply flow away. Instead, we followed Asher et al. (2019) and introduced a Pseudo-Atmospheric Pressure (PAP) forcing in the momentum equations. PAP is computed using the inverse barometer relationship and is added to the actual atmospheric pressure through the sea-surface pressure term (Fig. 2). Specifically, changes in atmospheric pressure create corresponding changes in water surface elevation (e.g., the bulge in sea level beneath an atmospheric low-pressure center). This can be approximated via the inverse barometer relationship, which modifies the sea surface pressure term in the momentum equations to reflect both the actual atmospheric pressure and PAP. The assimilation scheme is a continuous sequential approach, where the time-averaged differences between observed and simulated water levels are mapped onto the model grid using interpolation (Asher et al., 2019). The resulting adjustment of the total water elevation field then propagates through all other terms in the shallow water equations (Asher et al., 2019). In summary, the model was simulated using two different schemes. One simulation is defined as the “Open Loop” (OL) simulation; in other words, without any correction from the satellite observations. In this case, ADCIRC is simply forced by the wind and pressure from ERA5. For the other scheme, the “Data Assimilation” (DA) simulation, ADCIRC is corrected using satellite altimetry observations.

We used the KDTree algorithm (Bentley, 1975) to identify the closest node in the ADCIRC mesh to the satellite observation along the nadir track. During the simulation, modeled water levels at those points are corrected using the satellite water levels whenever an observation is available. We allowed a 1 hour ramp-up time for the DA simulation to reach the intended level to compensate for the shock received by the model due to these changes. This approach reduces the chances of any destabilization of the state of the model and maintains the corrected level throughout the simulation.

The simulations were then compared to NOAA tide gauge observations. Hourly output from both OL and DA was used to calculate Root Mean Square Error (RMSE), Mean Absolute Error (MAE), and Pearson Correlation Coefficient (CC) against NOAA-observed verified water levels. RMSE, MAE, and CC were used to evaluate the model performance with and without data assimilation. A total of 140 NOAA stations along the U.S. east coast were used for the evaluation. RMSE (Eq. 3), MAE (Eq. 4), and CC (Eq. 5) were calculated using the following equations:

3 $$RMSE=\sqrt{\frac{{\sum }_{i=1}^{N}{({SSH}_{\mathrm{i}}^{\mathrm{NOAA}} - {SSH}_{\mathrm{i}}^{\mathrm{Sim}})}^{2}}{N}}$$

4 $$MAE=\frac{{\sum }_{i=1}^{N}\left|{SSH}^{\mathrm{NOAA}} - {SSH}^{\mathrm{Sim}}\right|}{N}$$

5 $$CC= \frac{{\sum }_{i=1}^{N}({SSH}_{\mathrm{i}}^{\mathrm{NOAA}}- {SSH}_{\mathrm{mean}}^{\mathrm{NOAA}})({SSH}_{\mathrm{i}}^{\mathrm{Sim}}- {SSH}_{\mathrm{mean}}^{\mathrm{Sim}})}{\sqrt{{\sum }_{i=1}^{N}{({SSH}_{\mathrm{i}}^{\mathrm{NOAA}}- {SSH}_{\mathrm{mean}}^{\mathrm{NOAA}})}^{2}}\sqrt{{\sum }_{i=1}^{N}{({SSH}_{\mathrm{i}}^{\mathrm{Sim}}- {SSH}_{\mathrm{mean}}^{\mathrm{Sim}})}^{2}}}$$

where SSH^NOAA^ represents the water levels observed by NOAA tide gauge stations and SSH^Sim^ represents ADCIRC simulated water levels, both for OL and DA. *N* is the number of data points.

## Results

**Fig. 3:**
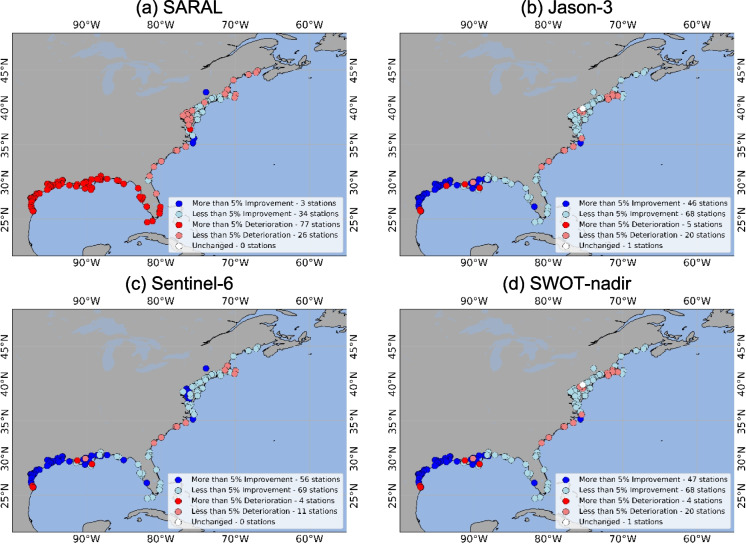
RMSE difference between OL and DA. When DA RMSE is greater than OL RMSE, the NOAA stations are marked as “Worsened,” based on if the improvement was more or less than 5%. When OL RMSE is greater than DA, the NOAA stations are marked as “Improved,” based on if the deterioration was more or less than 5%. The panels are represented by the four satellites as follows: **a** SARAL, **b** Jason-3, **c** Sentinel-6, **d** SWOT-nadir

Figure 3 shows the location and the number of stations where the RMSE improvement or the deterioration was more than 5%. The model performance improved at 37 stations in total (the DA performance at two of these is scrutinized later in this section) for SARAL, at 114 stations for Jason-3, 125 stations for Sentinel-6, and 115 stations for SWOT-nadir. DA deteriorated at 103 stations for SARAL, 25 stations for Jason-3, 15 stations for Sentinel-6, and 24 stations for SWOT-nadir.

**Fig. 4:**
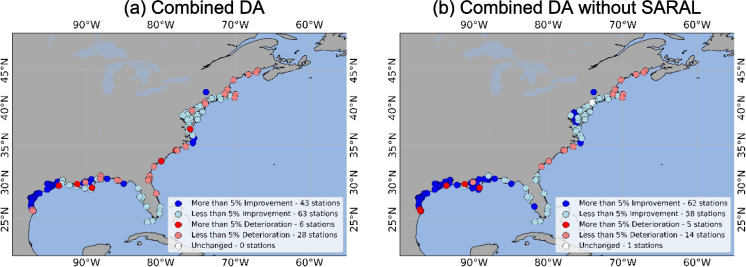
RMSE difference between OL and DA. **a** represents the DA with all four satellites considered, whereas **b** represents the DA where SARAL was excluded from the combined simulation, or in other words, only Jason-3, Sentinel-6, and SWOT-nadir were considered

For the combined data assimilation, we found that DA performed better than OL at 106 stations, while worsening the RMSE at a total of 34 stations (Fig. 4a). However, once we excluded SARAL (and only combined Jason-3, Sentinel-6, and SWOT-nadir), the total number of improved stations increases to 120, with only 19 stations showing worsening, as shown in Fig. 4b.

**Fig. 5:**
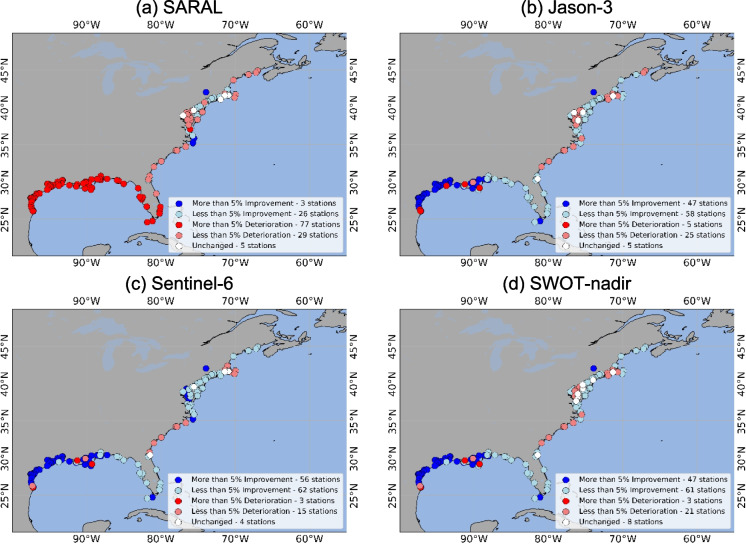
MAE difference between OL and DA. When DA MAE is greater than OL MAE, the NOAA stations are marked as “Worsened,” based on if the improvement was more or less than 5%. When OL MAE is greater than DA, the NOAA stations are marked as “Improved,” based on if the deterioration was more or less than 5%. The panels are represented by the four satellites as follows: **a** SARAL, **b** Jason-3, **c** Sentinel-6, **d** SWOT-nadir

For MAE, SARAL and SWOT-nadir observations improved model performance in total at 29 and 108 stations, respectively, whereas Jason-3 and Sentinel-6 showed improvements at 105 and 118 stations, respectively, in total (Fig. 5). SARAL worsened the MAE at a total of 106 stations, whereas Jason-3, Sentinel-6, and SWOT-nadir did it so at a total of 30, 18, and 24 stations, respectively. The MAE remained unchanged between DA and OL at 5, 5, 4, and 8 stations for SARAL, Jason-3, Sentinel-6, and SWOT-nadir, respectively.

**Fig. 6:**
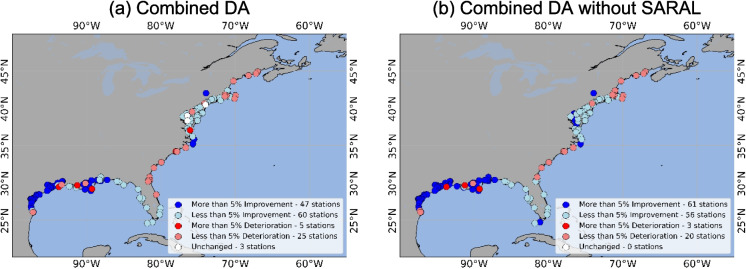
MAE difference between OL and DA, where **a** represents the DA with all four satellites considered, whereas **b** represents the DA where SARAL was excluded from the combined simulation, or in other words, only Jason-3, Sentinel-6, and SWOT-nadir were considered

Considering the combined simulation of all four satellites, a total of 107 stations saw improvements for MAE. At 30 stations, the MAE deteriorated. The performance at 3 stations remained unchanged (Fig. 6a). When the combined DA considered only Jason-3, Sentinel-6, and SWOT-nadir (i.e., without SARAL), MAE improved at a total of 117 stations. Only 23 stations saw deterioration in total (Fig. 6b). Similar to what was observed in Fig. 4 for RMSE, excluding SARAL from the combined DA increases the number of improved stations from 107 to 117 and reduces the number of stations that saw deterioration from 30 to 23.

**Fig. 7:**
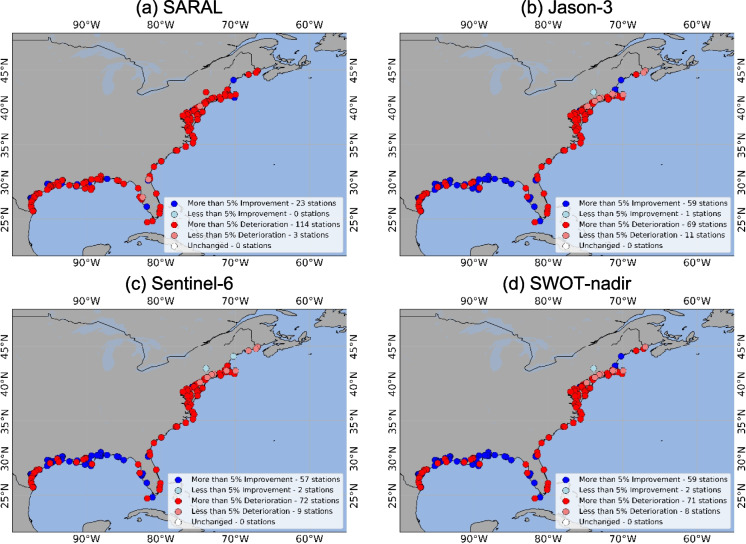
CC difference between OL and DA. When DA CC is greater than OL MAE, the NOAA stations are marked as “Improved,” based on if the improvement was more or less than 5%. When OL MAE is greater than DA, the NOAA stations are marked as “Worsened,” based on if the deterioration was more or less than 5%. The panels are represented by the four satellites as follows: **a** SARAL, **b** Jason-3, **c** Sentinel-6, **d** SWOT-nadir

For CC (Fig. 7), we observed that the correlation between NOAA tide gauge observations and DA simulations improved the most when DA was performed with Jason-3, Sentinel-6, and SWOT-nadir. With SARAL DA, a total of 23 locations saw improvements, whereas 117 locations saw deteriorations with correlation coefficient. For Jason-3, 60 stations improved, and 80 stations showed deterioration the correlation coefficient between NOAA tide gauge observations and DA simulations, compared to OL simulations. For Sentinel-6, a total of 59 stations showed improved performance regarding CC, and 81 stations deteriorated. For SWOT-nadir, a total of 61 station locations showed improved DA simulation performance regarding CC, and 79 stations experienced deterioration.

Next, we considered assimilating only satellite data flagged as being of good quality. For nadir altimeters, readings can be flagged as “bad” for several reasons, e.g., out-of-range tracker corrections, radar mispointing, land or ice contamination, signal saturation, or failure of the retracking algorithm. In this case, we have taken into account the flagged values from the “range_ocean_qual” variable of the altimeter products, which denotes the validity flag indicating the “good” or “bad” measurement of the ocean altimeter range. For this specific experimental case, we have only considered the data that have been flagged as “good” by the ocean altimeter range. In other words, if the nearest satellite observations to a mesh node were flagged as “bad”, it was replaced by the nearest “good”-quality data. The model performance with data flag consideration has been summarized in Table 1. More details about these results and plots can be found in the supplementary materials.

**Table 1:** OL and DA performance by station numbers when data quality flags were considered

Performance	SARAL			Jason-3			Sentinel-6			SWOT-nadir		
	RMSE	MAE	CC	RMSE	MAE	CC	RMSE	MAE	CC	RMSE	MAE	CC
Improved	37	33	25	114	105	60	125	118	59	115	110	60
Worsened	102	93	115	25	29	80	15	18	81	24	24	80
Unchanged	1	4	0	1	6	0	0	4	0	1	6	0

**Fig. 8:**
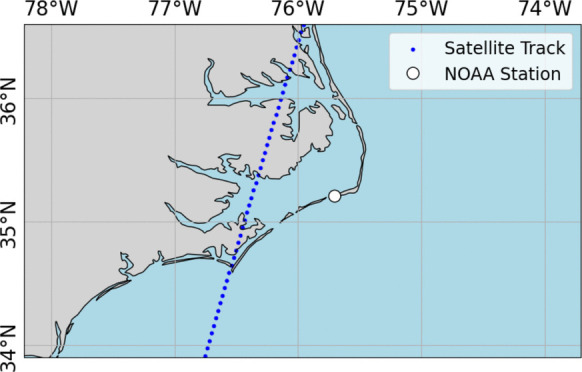
SARAL overpass on September 01, 2023, near NOAA Hatteras, NC station (NOAA: 8654467)

**Fig. 9:**
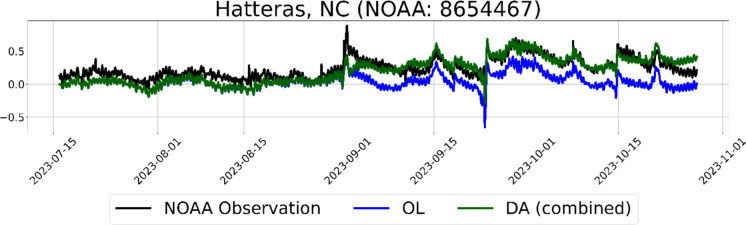
WL time series from July 16, 2023, to October 31, 2023, at Hatteras, North Carolina, depicting ADCIRC simulation performance improvement with SARAL overpass near the station

The improvement in the DA simulation is often contingent upon the presence of a satellite overpass near a simulation point. Especially during a surge event, the occurrence of a satellite overpass can notably capture correction appearing in the simulation. A closer inspection of two coastal floods that occurred during the simulation period provides insights into the influence of the frequency of satellite overpasses on the model performance. During the coastal flooding event of Tropical Storm Ophelia on the North Carolina coast, there was a SARAL overpass on September 01, 2023, as shown in Fig. 8. The time series of plain and corrected model simulations demonstrate significant improvement of model performance correlated with that overpass at Hatteras station (Fig. 9). The time series captures the improvement immediately after the SARAL overpass occurred over an ADCIRC node close to the NOAA Hatteras tide gauge (8654467).

**Fig. 10:**
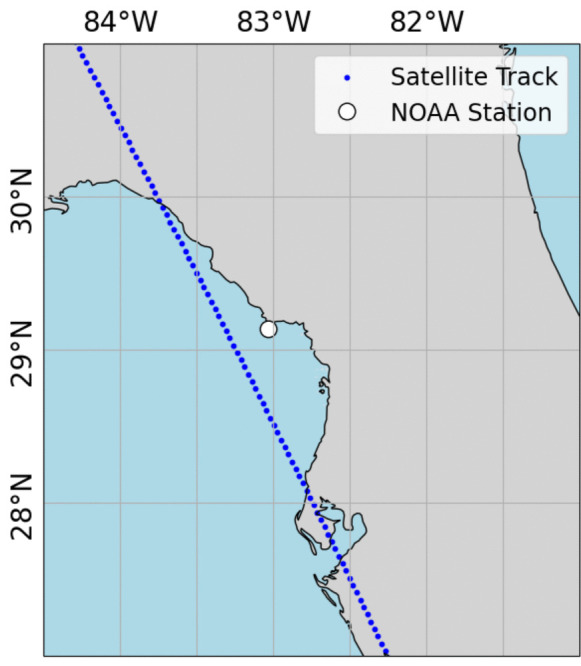
Sentinel-6 overpass on August 30, 2023, near NOAA Cedar Key, FL station (NOAA: 8726520)

**Fig. 11:**
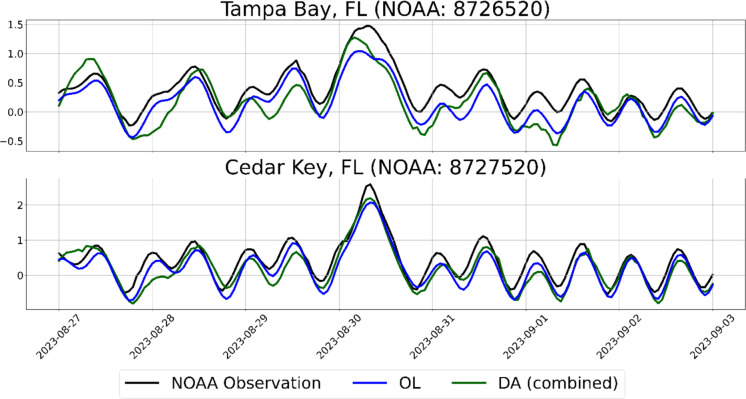
WL time series from August 27, 2023, to September 03, 2023, at Cedar Key and Tampa Bay, Florida stations depicting ADCIRC simulation performance improvement with Sentinel-6 overpass near the stations during Hurricane Idalia

Similarly, an overpass from Sentinel-6 was available on August 28, 2023, near the Cape Florida station, when Hurricane Idalia made landfall in the Florida Bay and caused storm surge flooding (Fig. 10). Sentinel-6 clearly pushes the DA model simulation closer to the observed water level time series during the surge period, as shown in Fig. 11. The DA simulation reaches closer to the levels observed by the Tampa Bay (8726520) and Cedar Key (8727520) stations in Florida around August 28 in the time series.

## Discussion

The first set of questions aimed to be answered in this study is: Are satellite altimetry data capable of improving ADCIRC performance for estimating CWL? If so, what is the extent of such an improvement? To answer these questions, we performed two different simulations, one without any satellite assimilation and another including satellite assimilations. Five DA simulations were run: four simulations for each of the four satellites (SARAL, Jason-3, Sentinel-6, and SWOT) and one that combined all satellites. We further analyzed the effects of satellite data quality flags on the performance of the DA runs. ADCIRC simulations with satellite corrections were compared to the simulation without correction. First, RMSE and MAE were calculated between NOAA-observed WL along the U.S. East Coast and the OL, and then again between NOAA-observed WL and the DA. RMSE, MAE, and CC were calculated for the study period between August 01, 2023, and October 31, 2023, with hourly CWL outputs.

When it comes to assimilating altimetry data from multiple satellite sources into a hydrologic-hydrodynamic model, Wongchuig et al. (2024) achieved more than 30% error reduction in discharge and water level estimations by applying more robust data assimilation techniques compared to direct insertion in a river basin. Directly assimilating altimetry data from a single satellite mission is also capable of improving water level estimations by more than 10% in 1-D hydrodynamic river model when simulated in large river basins (Schneider et al., 2018). Despite traditional nadir along-track altimetry data being regarded as below par, several previous studies have demonstrated their utility in coastal areas. Roblou et al. (2011) argued that the new generation of satellite altimetry missions is better suited for applications in coastal modeling. The results shown in our study confirm the value of merging satellite altimetry data into a hydrodynamic model. The improvement in model performance can be due to several reasons. Asher et al. (2019) demonstrated that mesoscale and sub-mesoscale dynamics are difficult to simulate in coastal models. Nevertheless, these phenomena are detected by satellite altimetry whenever an overpass is available, as demonstrated by the two case studies analyzed in our work. A natural progression of this work is to analyze the effect of the unresolved drivers (e.g., AMOC) in the modeling process, which are otherwise detected by new generation satellite observations, by applying robust filtering techniques, e.g., low-pass filters, to long-term water level records. Future studies are encouraged to investigate the applicability of a more suitable tide model for coastal areas, which can potentially improve the simulation accuracy (Stammer et al., 2014).

It should be noted that simulations are improved through DA during surge events only when there was an overpass close to a station immediately before the surge event. It is likely that such improvements occurred in other parts of the domain. However, the performance of the model performance can only be assessed where NOAA tide gauges are located. Furthermore, due to the nature of the DA method (i.e., based on PAP forcings), the model update remains in memory for the remainder of the simulation. It can be argued that the assimilation scheme is more suitable for long-term bias corrections (e.g., low-frequency oceanic phenomena) in coastal water level simulations. Although on multiple occasions the DA-induced improvement was minimal, such enhancement can be critical for storm surge flood forecasting and early warning systems, once the simulated WL is translated into flood inundation mapping outputs.

The capabilities of satellite observations to improve model performance can be traced to the individual specifications of each satellite. In our experiment, ADCIRC simulations perform better when corrected with Jason-3, Sentinel-6, and SWOT observations, compared to when corrected with SARAL products. SWOT-nadir observations, which operate with larger footprint (1–10 km) compared to those of SARAL, improved the OL simulations at the largest number of stations. One possible explanation for SARAL’s limited performance could be its coarse temporal resolution. SARAL has an orbital repeat frequency of 35 days, compared to the 10-day frequency of Jason-3 and Sentinel-6, and 4-day frequency of SWOT. Higher temporal resolution allows for better detection of rapidly evolving coastal phenomena and for more spatial coverage leading to more observations at more locations. SARAL’s sun-synchronous orbit also limits it to detecting the solar constituents of the tide, preventing it from identifying fluctuations caused by other constituents. Furthermore, our study underscores the importance of satellite data quality flags. Model performance deteriorated in all four satellite altimetry mission cases when the data points flagged as “bad” were not assimilated. It can be argued that the omission of flagged observations in nearshore areas compelled the assimilation scheme to replace those observations with the closest unflagged observations, which ultimately contributed to the performance deterioration since the replaced observations were not close enough. Despite being flagged, the along-track nadir altimetry data can be useful for operational purposes as the standard of the quality flags also degrades near the coastline occasionally (Andersen & Cheng, 2013; Cheng et al., 2012). Further studies should be performed to determine a threshold for the proximity of satellite observation points to NOAA tide gauge observation stations for identifying improvements in model performance in estimating CWL.

Despite improving simulation performance at a few stations, the nadir-only satellite altimetry missions have reduced model performance at other stations, underscoring the shortcomings of nadir altimetry in coastal zones. Presumably, the inadequate spatial coverage of nadir-only altimetry makes it difficult for satellite altimetry missions to accurately capture the rapidly fluctuating phenomena. The factors affecting CWL are highly localized, and most of the current altimetry missions are not designed to capture those localized fluctuations. It is likely that such dynamics will be captured by wide-swath altimetry missions, e.g., SWOT, and that merging SWOT swath altimetry data will further improve coastal hydrodynamic models.

It cannot be ruled out that the assimilation scheme used for merging satellite data into the model played a role in the deterioration of model performance at a handful of stations. Bhuiyan et al. (2024) showed that instantaneous changes to water level unsupported by the model parameters can produce a “shock” to the model. Additionally, it can quickly nullify the effect of merging satellite observations. To address this issue, we used the Dynamic Water Level Correction scheme in ADCIRC, which allows the model to ramp up to the levels observed by the satellites, reducing the effects of sudden changes. However, due to the intervals of satellite overpasses, this study limited the lowest ramp-up time to 1 h. Such a short ramp up time may have contributed to the deterioration of model performance in a few locations. The degraded CC of the DA simulation, when compared to the OL, appears to be an artifact of the constrained ramp-up time. Given the nonstationary nature of coastal water levels, Pearson CC is highly sensitive to phase shifts. We hypothesize that the shortened ramp-up period introduces such a temporal lag in the simulated time series. This misalignment may degrade the CC score given that the correct water level magnitudes are predicted at incorrect times, even as other magnitude-based error metrics (which are less sensitive to phase errors) demonstrate overall improvement. This can also be partly explained by the uncertainty associated with the proliferation of information from one node to adjacent nodes. The applied model scheme uses an error covariance matrix to communicate the change of water level due to satellite observations to the adjacent nodes, where the uncertainties involving the error  covariance matrix can lead to less-than-ideal WL estimation.

## Conclusion

This study sets out to evaluate the efficiency of using nadir-only satellite altimetry data from multiple satellite missions to improve CWL simulations produced by the ADCIRC hydrodynamic model. Our findings demonstrate that satellite observations have the potential to enhance the accuracy of such models, especially when a satellite overpass occurs close to a coastal flooding event. ADCIRC simulations were merged with altimetry data from four satellite missions (SARAL, Jason-3, Sentinel-6, and SWOT-nadir). Results demonstrated that SWOT-nadir, with high spatial resolution and a more frequent revisit period, provided the greatest improvement to CWL simulations. When combined, satellite altimetry observations can further improve model performance when compared to individually merging altimetry data. The study underscores the potential of the conventional nadir-only along-track satellite data in capturing fine-scale processes that dominate coastal dynamics, while suggesting the implementation of similar schemes using observations from state-of-the-art wide-swath altimetry products.

Nevertheless, the study is limited by the complexity and difficulty of assimilating satellite altimetry data into hydrodynamic models. The improvements in model performance achieved through DA were not ubiquitous. Several locations experienced little to no improvement, and even experienced degradation after DA. These mixed outcomes underline known limitations of nadir altimetry in coastal zones: coarse spatial coverage, long repeat periods, uncertain geophysical corrections nearshore, and sensitivity to vertical land motion. Additionally, our choice of a relatively simple DA scheme makes assumptions about the propagating spatial WL information and ramp-up time. Such caveats highlight that this approach is not an all-encompassing solution. Previous studies have noted that operational storm-surge models already assimilate altimetry and other observations, and that altimetry assimilation is a major but routine component of storm surge forecasting.

These findings point to several directions for future work. First, exploring more sophisticated DA schemes, e.g., EnKF or 4D-Var, may help incorporate satellite products more realistically into the model and reduce the risk of over-correction. Second, incorporating wide-swath altimetry, which offers denser spatial coverage compared to nadir altimetry, or applying custom retracking algorithms to altimeter readings that are better suited for observations near coastal areas, may attenuate the sampling limitations of nadir missions. Third, jointly assimilating altimetry products with other data sources (e.g., tide gauge data) could address existing biases in both the storm surge model and the satellite data. Finally, comprehensive sensitivity studies are needed to determine how DA ramp-up time, quality-flag thresholds, and proximity to observation points influence the assimilation process. Altimetry assimilation has the potential to make an effective contribution to operational coastal flood forecasting and to further our understanding of coastal hazards.

## Supplementary Information

Below is the link to the electronic supplementary material.

ESM 1 (DOCX 610 KB)

## Data Availability

Name of software: ADCIRC (ADvanced CIRCulation)Documentation: Detailed documentation for application HPC installation, local installation, testing, deployment, and use of software can be found at https://adcirc.org. [https://adcirc.org/](https:/adcirc.org). Data repository: The result data from one of the experiment simulations are publicly available. Due to the size of each result file (~9GB each, total 10 files), the rest of the result files will be shared if requested. The data archive can be found at 10.5281/zenodo.18446697 Size of the archive: 9GB.
