# Supplementary material for: Improving coastal water level estimation by merging nadir-only satellite altimetry data into a hydrodynamic model
Source: Environ Monit Assess. 2026 Mar 14;198(4):312. doi: 10.1007/s10661-026-15166-8 (PMC12988993; doi:10.1007/s10661-026-15166-8)
Supplement: Supplementary file 1 — (DOCX 610 KB) [file 10661_2026_15166_MOESM1_ESM.docx]

We observed an improvement (with respect to the OL) of more than 5% in RMSE at 3 stations for SARAL and 12 stations for Sentinel-6, whereas no stations showed an improvement for Jason-3 or SWOT-nadir (**Figure 1**). However, a deterioration in model performance of more than 5% was seen at 77 stations for SARAL and 1 station for Jason-3, while Sentinel-6 and SWOT-nadir did not show any performance decline at any station. When compared to the original DA in which quality flags were not considered, SARAL saw small improvements for two more stations and 3 fewer stations saw a small deterioration. For Jason-3 when quality flags were considered, 28 more stations saw an improvement of more than 5%. For Sentinel-6, both cases (quality flags considered/not considered) showed the same results for all the stations. However, for SWOT-nadir, 4 fewer stations saw more than 5% improvement compared to when quality flags were not considered. The number of stations seeing more than 5% deterioration remained the same for both cases for SWOT-nadir. Performance remained unchanged for 2 fewer stations when quality flags were considered.

| (a) SARAL  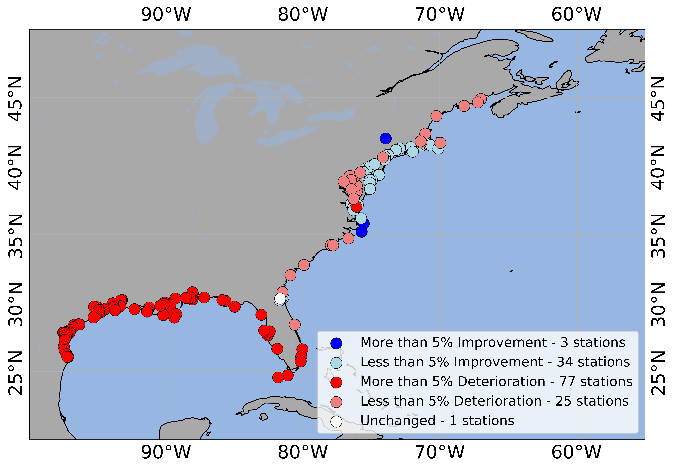 | (b) Jason-3  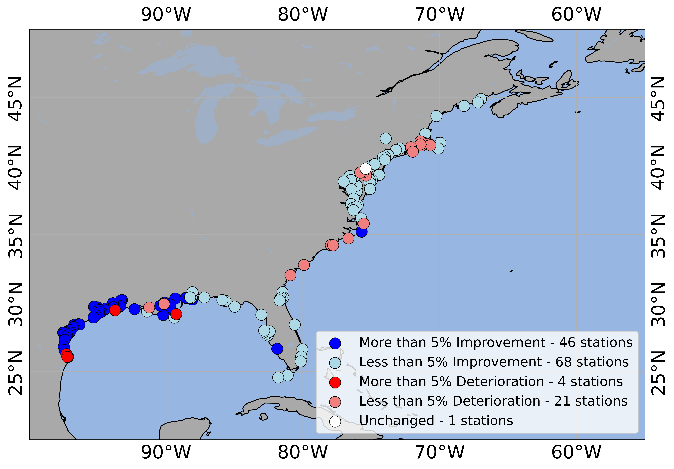 |
| --- | --- |
| (c) Sentinel-6  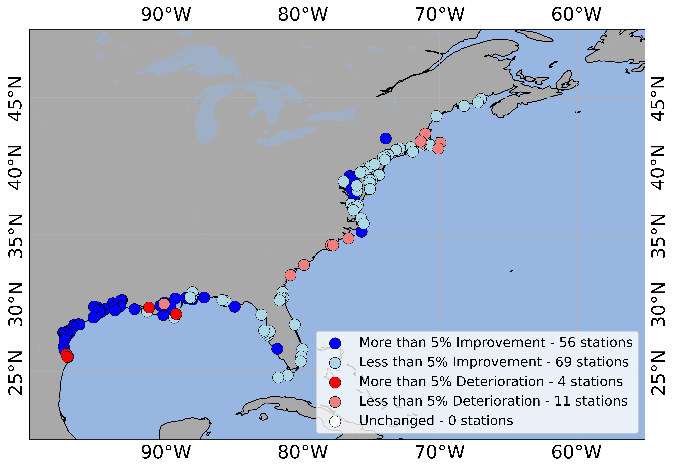 | (d) SWOT-nadir  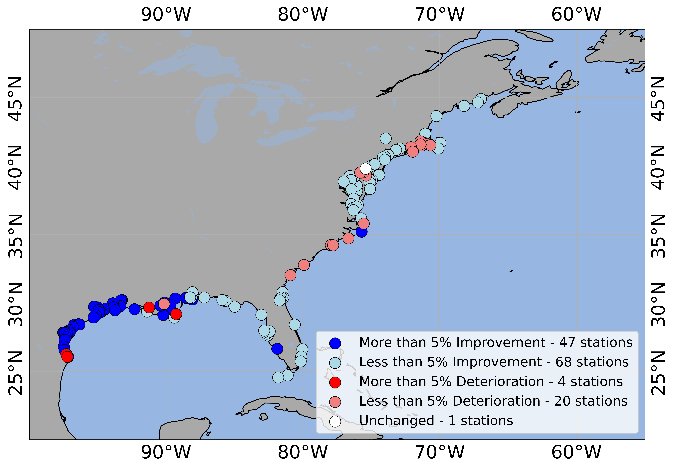 |

Figure 1 RMSE difference between OL and DA with satellite data quality flags considered. When Corrected Simulation RMSE is greater than OL RMSE, the NOAA stations are marked as "Improved", based on if the improvement was more or less than 5%. When OL RMSE is greater than DA, the NOAA stations are marked as “Worsened”, based on if the deterioration was more or less than 5%. The panels are represented by the four satellites as follows – (a) SARAL, (b) Jason-3, (c) Sentinel-6, (d) SWOT-nadir

Further analysis of the impact of removing flagged observations from the assimilated altimetry data on MAE (**Figure 2**) revealed that model performance improved by more than 5% at 3 stations for SARAL and 11 stations for Sentinel-6. In contrast, Jason-3 and SWOT-nadir showed no stations with an improvement greater than 5%. A smaller improvement (less than 5%) was observed at 28, 71, 82, and 115 stations for SARAL, Jason-3, Sentinel-6, and SWOT-nadir, respectively. A deterioration of more than 5% was noted at 79 stations for SARAL and 1 station for Jason-3, while Sentinel-6 and SWOT-nadir did not show performance deterioration of over 5% at any station. Additionally, model performance deteriorated by less than 5% at 34, 22, 38, and 21 stations for SARAL, Jason-3, Sentinel-6, and SWOT-nadir, respectively. Finally, no significant performance changes between OL and DA (with data quality flags considered) were observed at 3, 53, 15, and 11 stations for SARAL, Jason-3, Sentinel-6, and SWOT-nadir, respectively. When comparing both cases (quality flag considered/not considered) for each satellite individually with MAE, SARAL performed the same for both cases. The number of stations seeing no performance change when flags were considered lessened by 2. For Jason-3, the number of stations seeing more than 5% improvement and deterioration remained the same for both cases. The number of stations seeing no change in performance increased by 31 when quality flags were considered. For Sentinel-6, 11 more stations saw more than 5% improvement when flags were considered, when 1 more station saw more than 5% deterioration for the same case. Performance remained unchanged for 7 fewer stations for the latter case.

For SWOT-nadir, 4 fewer stations showed more than 5% improvement when quality flags were considered, whereas performance deterioration by more than 5% remained the same for both cases. Performance remained unchanged for 2 fewer stations when quality flags were considered.

| (a) SARAL  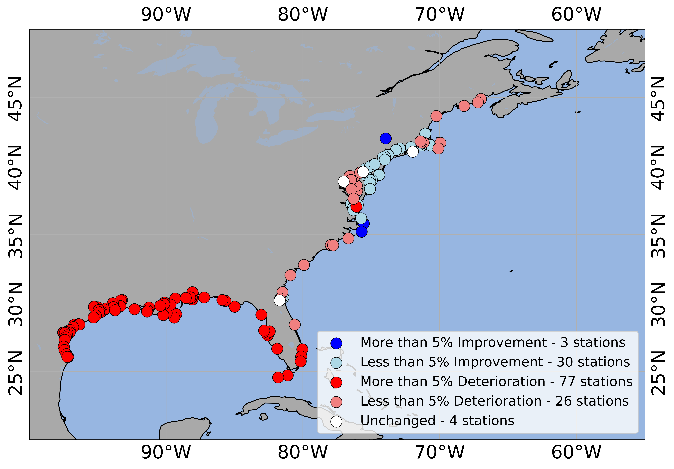 | (b) Jason-3  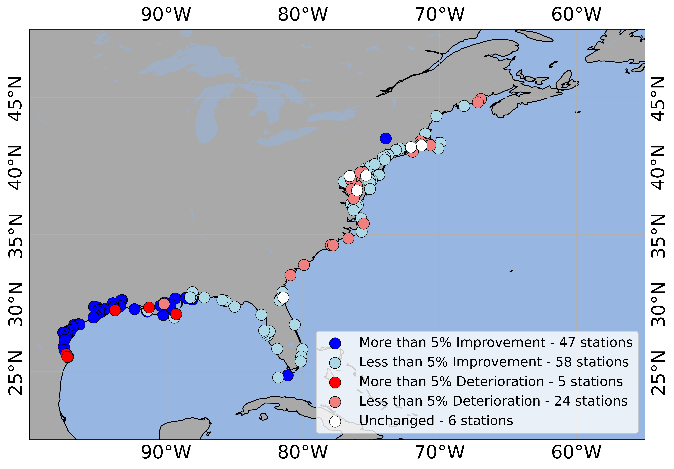 |
| --- | --- |
| (c) Sentinel-6  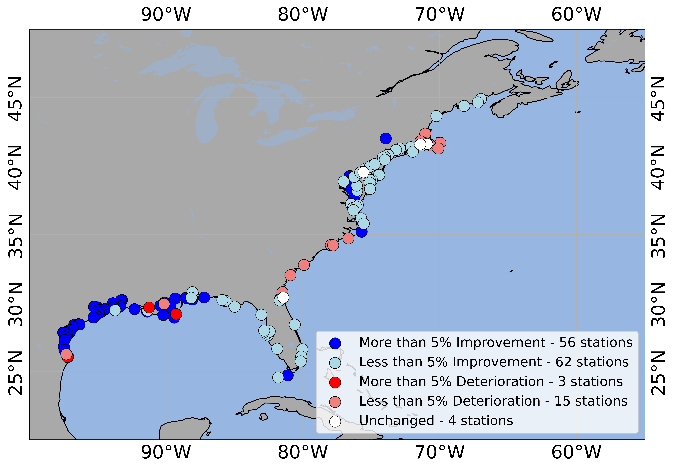 | (d) SWOT-nadir  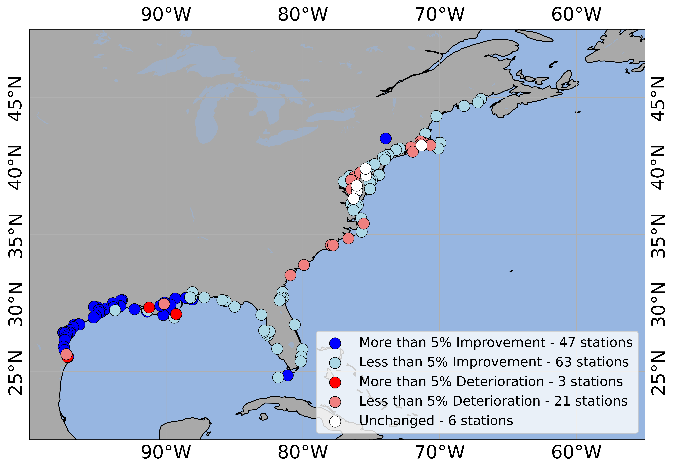 |

Figure 2 MAE difference between OL and DA with satellite data quality flags considered. When Corrected Simulation MAE is greater than OL MAE, the NOAA stations are marked as "Improved", based on if the improvement was more or less than 5%. When OL MAE is greater than DA, the NOAA stations are marked as “Worsened”, based on if the deterioration was more or less than 5%. The panels are represented by the four satellites as follows – (a) SARAL, (b) Jason-3, (c) Sentinel-6, (d) SWOT-nadir

| (a) SARAL  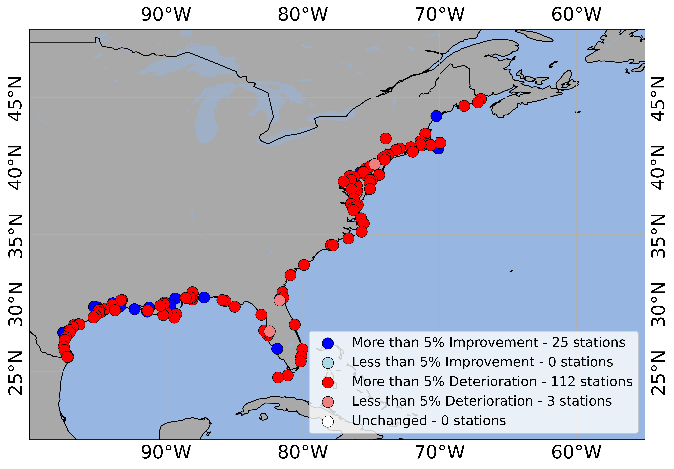 | (b) Jason-3  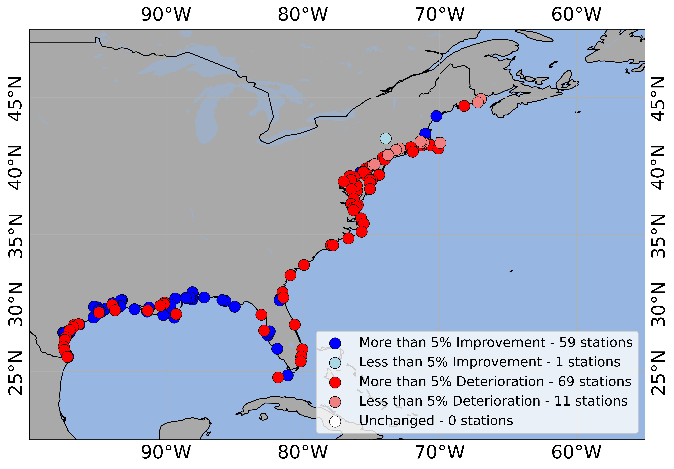 |
| --- | --- |
| (c) Sentinel-6  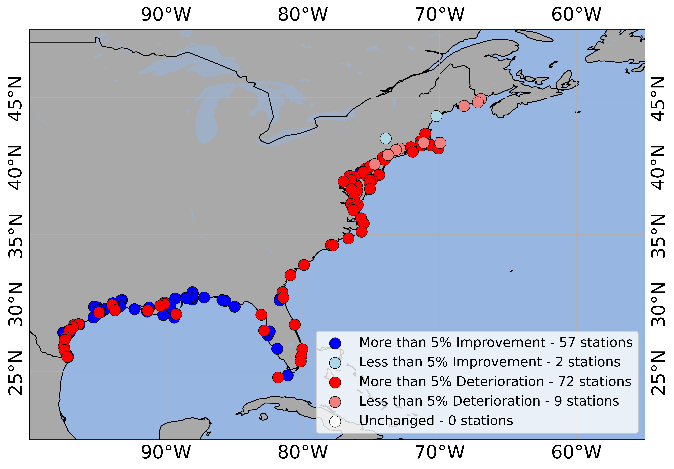 | (d) SWOT-nadir  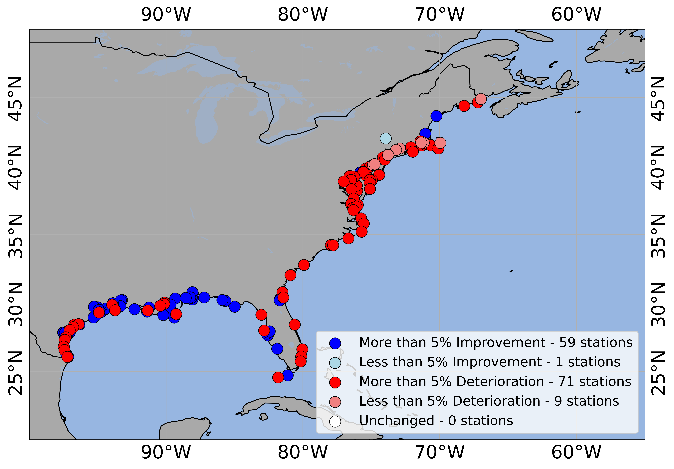 |

Figure 3 CC difference between OL and DA with satellite data quality flags considered. When Corrected Simulation CC is greater than OL CC, the NOAA stations are marked as "Improved", based on if the improvement was more or less than 5%. When OL CC is greater than DA CC, the NOAA stations are marked as “Worsened”. The panels are represented by the four satellites as follows – (a) SARAL, (b) Jason-3, (c) Sentinel-6, (d) SWOT-nadir
